# Disulfidptosis-associated LncRNAs index predicts prognosis and chemotherapy drugs sensitivity in cervical cancer

**DOI:** 10.1038/s41598-023-39669-3

**Published:** 2023-08-01

**Authors:** Li Liu, Jun Liu, Qianbao Lyu, Jinzhi Huang, Yuanfeng Chen, Cuiyi Feng, Yaoyao Liu, Fukun Chen, Zhouyan Wang

**Affiliations:** 1 Department of Gynecology, Shunde Women and Children’s Hospital (Maternity and Child Healthcare Hospital of Shunde Foshan), Guangdong Medical University, No.1 Baojian Road, Shunde District, Foshan, 528300 Guangdong China; 2 Department of Obstetrics, Pingxiang Maternal and Child Health Hospital, Pingxiang, 337000 Jiangxi China; 3 Geneplus-Beijing Institute, Beijing, 10000 China; 4 Department of Pharmacy, Shunde Women and Children’s Hospital (Maternity and Child Healthcare Hospital of Shunde Foshan), Guangdong Medical University, Foshan, 528300 Guangdong China

**Keywords:** Cancer, Computational biology and bioinformatics, Genetics, Biomarkers, Oncology

## Abstract

Disulfidptosis is a newly discovered form of cell death. Not yet clearly classified as programmed cell death or accidental cell death. This study aimed to create a novel disulfidptosis-related lncRNA index (DLI) that can be used to predict survival and chemotherapy drugs sensitivity in patients with cervical cancer. First of all, we found lncRNAs associated with disulfidptosis between cervical cancer tissues and normal tissues. By LASSO-Cox analysis, overlapping lncRNAs were then used to construct lncRNA index associated with disulfidptosis, which can be served to predict the prognosis of patients with CC, especially the chemotherapy drugs sensitivity. ROC curves and PCA based on DLI and clinical signatures were developed and demonstrated to have good predictive potential. In addition, differences in immune cell subset infiltration and differences in immune checkpoint expression between high-DLI and low-DLI groups were analyzed, and we investigated the relationship between the DLI and tumor mutation burden (TMB). In summary, we constructed a lncRNA prediction index associated with disulfidptosis. This has important clinical implications, including improving the predictive value of cervical cancer patients and providing a biomarker for cervical cancer guiding individualized treatment.

## Introduction

In recent years, a remarkable surge in global cancer incidence and associated mortality rates has been reported. Cervical cancer (CC) ranks fourth in terms of the highest mortality and incidence rates among women globally^1,2^. The global age-standardized incidence of CC varies substantially among countries, with reported rates ranging from fewer than 13 cases to 1 case per 70,000 women. Noteworthy, CC is the chief cause of cancer-related deaths among women in low- and middle-income countries^3^. It is widely known that high-risk HPV strains are associated with CC. C While HPV is necessary for the transformation of cervical epithelial cells, other co-factors and molecular procedures also the occurrence of CC^4,5^. Therefore, the exploration of novel potential biomarkers and underlying mechanisms is important for predicting the prognosis and improving the treatment of CC.

Cell death is a crucial process for human health. In 2018, the Cell Death Nomenclature Committee classified this process into accidental cell death (ACD) and regulatory cell death (RCD). RCD, also known as programmed cell death (PCD), is a fully physiological form of cell death^6^. It plays an essential role in the development of an organism, acts as a host defense mechanism against pathogens and maintains homeostasis. However, excessive activation of the PCD pathway exerts detrimental effects and may lead to disease development^7^. At least 12 types of PCD that are implicated in the development of malignant tumors. In glucose-deficient *SLC7A11*^−high^ cancer cells, the excessive accumulation of disulfide molecules causes abnormal disulfide bonding between actin cytoskeletal proteins, thus disrupting their organization and eventually leading to the collapse of the actin network and cell death. This effect, known as disulfidptosis, effectively inhibits the growth of malignant tumors with any apparent toxicity to normal tissues^8,9^. This process of disulfidptosis involves glycogen accumulation, energy metabolism, mitochondrial respiration and disulfide regulation. The genes responsible for regulating these processes have been identified from published literature. For instance, *GYS1* induces glycogen accumulation^10^; *LRPPRC* affects mitochondrial autophagy by regulating energy metabolism^11^; *NDUFA11* and *NDUFS1* maintain the mitochondrial structure and function^12^; *NUBPL* is involved in the mitochondrial respiratory chain^13^; *OXSM* is involved in glycogen regulation^14^; *NCKAP1*, *RPN1*, *SLC3A2*, and *SLC7A11* are involved in the regulation of disulfidptosis^8,9^. Therefore, further investigations on disulfidptosis, the genes affecting glucose and lipid metabolism, and the underlying mechanism of disulfidptosis will aid in the identification of potential novel targets of this disease.

Non-coding RNAs (ncRNAs) represent a novel class of transcripts. That, although mostly not translated into proteins, play crucial roles in various cellular and physiological functions^15^. Among them, long ncRNAs (lncRNAs) are implicated in cancer development, indicated by their mutations and dysregulated expression. lncRNAs can promote tumorigenesis and metastasis, while also possessing tumor-suppressive and pro-cancer functions^16^. As such, lncRNAs hold immense promise as novel biomarkers and therapeutic targets for cancer^17^. For instance, the lncRNA SNHG1 is involved in the migration and invasion of CC^18^. Considerably linked with tumor size, FIGO stage, and lymph node metastasis, The lncRNA *FALEC* has shown substantially elevated levels in the plasma of CC patients. Additionally, overexpression of lncRNA *FALEC* promotes HeLa cell invasion and proliferation^19^. However, to date, the specific disease targets and underlying mechanisms through which lncRNA regulate disulfidptosis-related genes and affect the prognosis of CC by means of disulfidptosis remain unexplored. We extracted RNA sequencing data of patients with CC from publicly accessed databases and used bioinformatics methods to screen for lncRNAs associated with disulfidptosis related-genes (such as *GYS1* etc.) that have predictive prognostic significance. This study intended to identify ncRNA-based biological targets for predicting CC prognosis, discover potential chemotherapeutic drugs targeting ncRNAs, and unravel the mechanisms underlying disulfidptosis in CC.

## Materials and methods

### Downloading and organizing raw data

Transcriptomic data, clinicopathological data, copy number variation (CNV) data, and single nucleotide variation (SNV) data of patients with CC (304 tumor tissues and 3 normal tissues) from The Cancer Genome Atlas (TCGA) database (https://www.cancer.gov/). The Perl programming language (version 5.32.1.1) was used to organize data for subsequent analysis. The CNV profile of disulfidptosis-associated genes in CC was assessed by constructing a lollipop plot, and the R package “RCircos” was utilized to map the variant loci on the chromosome. Additionally, waterfall plots were constructed to detect the mutations existing in disulfidptosis-associated genes in CC. Among 289 cervical cancer samples, we screened these ten genes for somatic mutations in 29 samples and counted the somatic mutation frequencies.

### Acquisition of disulfidptosis-associated lncRNAs and establishment of disulfidptosis-associated lncRNA index

Details of ten disulfidptosis-related genes were manually compiled from the literature. Pearson correlation analysis was performed to evaluate the relationship between disulfidptosis-related genes and lncRNAs, which was visualized using Sankey diagrams. The criteria for deducing the association were |R^2^|> 0.4 and p < 0.001. Univariate COX analysis was performed to screen for prognosis-related lncRNAs. Based on clinicopathological traits, patients with CC in the TCGA-CESC dataset were randomly split into a training cohort and a testing cohort in a 1:1 ratio. LASSO and multivariate COX regression were used for the training cohort to further refine the selection of the lncRNAs for model construction. Internal validation was subsequently performed in the testing cohort as well as in the entire TCGA dataset (TCGA cohort). The best prediction model was determined using the penalty parameter calculated through 1000-fold cross-validation with a p-value of 0.05.

The final formula for establishing the disulfidptosis-associated lncRNA index (DLI) is as follows:

$${\text{DLI}} = ({\text{coef}}_{{{\text{ZSCAN16}} - {\text{AS1}}}} \times {\text{ exp}}_{{{\text{ZSCAN16}} - {\text{AS1}}}} ) + ({\text{coef}}_{{{\text{AC}}083799.1}} \times {\text{ exp}}_{{{\text{AC}}083799.1}} ) + ({\text{coef}}_{{{\text{AL}}0{217}07.6}} \times {\text{ exp}}_{{{\text{AL}}021707.6}} ) + ({\text{coef}}_{{{\text{LINC}}02356}} \times {\text{ exp}}_{{{\text{LINC}}02356}} ) + ({\text{coef}}_{{{\text{AC}}023043.1}} \times {\text{ exp}}_{{{\text{AC}}023043.1}} ),$$

where coef_lncRNA_ represents the link between a lncRNA and the survival of patients with CC. and exp_lncRNA_ represents the expression level of that particular lncRNA.

In addition, we validated the expression differences of the lncRNAs screened using the Wilcox test to test whether there were expression differences between normal and tumor tissues in the TCGA dataset.

### Characterization of DLI

To assess the predictive value of the DLI in CC, we conducted validation analyses in each of the three cohorts: TCGA cohort, training cohort, and testing cohort. In the TCGA cohort, patients with CC were categorized into low-risk (< median) or high-risk (≥ median) groups based on median scores of DLI (Risk). Kaplan–Meier curves depicting overall survival (OS) and progression-free survival (PFS) were generated using the R packages “survminer” and “survival,” and the differences between the risk groups were calculated. Scatter plots, risk curves, and heatmaps were constructed to confirm the distribution of risk values among patients in different risk groups and to deduce the risk of CC-associated death.

### Validation of the predictive ability of DLI

Univariate and multivariate COX regression analyses were performed to evaluate the independence of DLI relative to other clinical characteristics. Receiver operating characteristic curves (ROC) were generated to assess the sensitivity and specificity of prognostic predictive value of the DLI when compared with other clinicopathological characteristics in patients with CC.

### PCA analysis and nomoscore calculation

Principal component analysis (PCA) analysis was employed to compare the risk definition and spatial distribution of patients based on various genetic classifications. This analysis facilitates the clinical utilization of the DLI and provides a comprehensive evaluation of patient prognosis. We integrated the prognostic characteristics (age, grade, and T, N, M) of patients with CC to calculate the predictive power on 1-, 3- and 5-years OS. Furthermore, multivariate Cox regression and stepwise regression analysis were performed to identify independent predictors Column plots were constructed, and nomoscore was calculated, which serves as a predictive tool for the outcomes of patients with CC. A nomogram was developed using the R package "regplot".

### GO analysis and GSEA analysis

We used gene ontology (GO) to analyze the differential genes between high- and low-DLI groups using the "clusterProfiler" R package. In addition. We used GSEA software (version 6.2) to perform a gene set enrichment analysis (GSEA) and identify the primary pathways of action in the high- and low-DLI groups. The statistical significance of the screen was set at P < 0.05 and the false discovery rate (FDR) q < 0.05.

### Analysis of the immune infiltration of the DLI and IPS score

We evaluated immune cell infiltration in TCGA samples using seven methods. The “GSVA” and “GSEABase” R packages are used to calculate differences in immune function between different risk groups. A box plot was also used to show the differences in immune checkpoint and immune cell infiltration between high- and low-DLI groups. Immunophenoscore (IPS) obtained from The Cancer Immunome Atlas (TCIA) database (https://tcia.at/home).

### Tumor mutation burden landscape and chemotherapy drug sensitivity prediction

The "maftool" package was used to create waterfall plots showing differences in tumor mutation burden (TMB) between high- and low-risk groups. The Wilcoxon test was used to analyse survival differences between high- and low-mutation groups, and for joint survival analysis with different DLI groups. KM curves were used to visualise survival analysis.

Drug sensitivity was calculated using the "oncopredict" software package. This package was developed by Maeser et al.^20^ for drug sensitivity prediction. The IC_50_ value represents the sensitivity of the chemotherapeutic drug in the cell lines of this cancer type. Higher IC50 values mean that the drug is less sensitive and a higher dose is required to achieve the same efficacy. It fits the gene expression profile of a tissue to the half-maximal inhibitory concentration (IC_50_) of a cancer cell line to the economics of Drug Sensitivity in Cancer (GDSC). Download details from GDSC on molecular indicators of drug sensitivity and response in cancer cells^20^ to generate models that can be applied to CC transcriptomics data. The data from our high- and low-DLI groups were correlated with the expression profile data from GDSC, and then unpaired t-tests were used to compare the differences in IC50 of chemotherapeutic agents between the high and low DLI groups. A sensitivity score was then performed to forecast the IC50 for each drug's half-maximal inhibitory concentration in CC patients.

### Statistical analysis

Software used in this study included R language and PERL, GSEA software (version). p < 0.05 (marked with *) was considered statistically significant.

## Results

### Differential expression and variation landscape of disulfidptosis-associated genes in patients with CC

**Figure 1:**
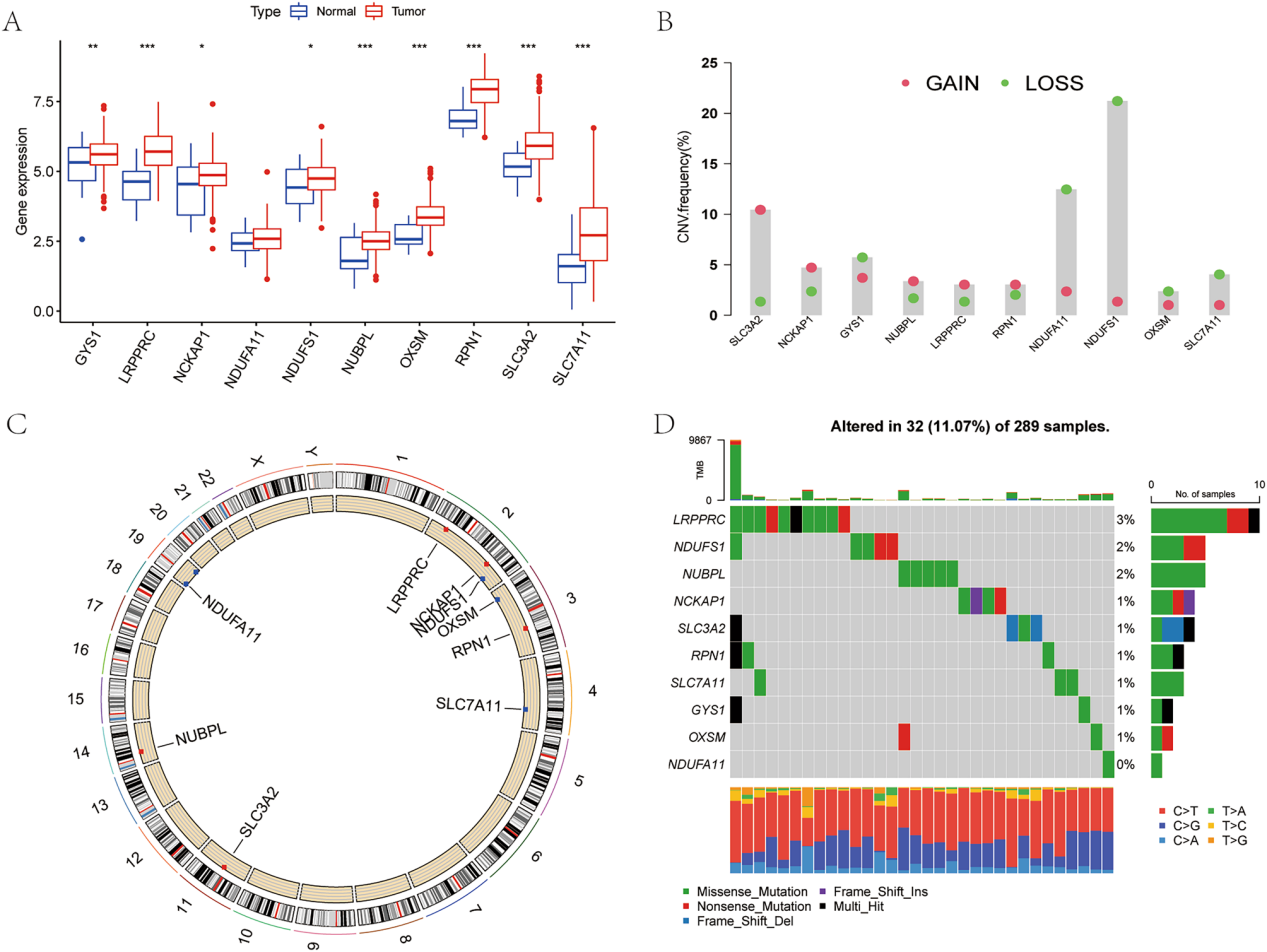
Differences in gene expression and variation landscape associated with disulfidptosis. (**A**) Differences in the expression of disulfidptosis-related genes between cervical cancer tissue and normal tissues. (**B**) The CNV landscape of disulfidptosis-related genes in TCGA-CESC datasets. (**C**) The site of CNV in disulfidptosis-related genes on chromosomes. (**D**) Somatic mutation landscape of disulfidptosis-related genes.

We analyzed the expression differences for the ten disulfidptosis-related genes between CC tumor tissues and normal tissues. Our findings revealed that all ten genes had upregulated expression in tumor tissues (Fig. 1A). Except for *NDUFA11*, the remaining nine genes showed significant differences in expression. Additionally, we investigated the CNV of the 10 genes in CC and observed that the CNVs were predominantly deletions. Notably, *NDUF11* and *NDUFS1* showed the highest number of copy number deletions (Fig. 1B), while *SLC3A2* showed the most substantial increase in DNA copy number. The gene chromosomal loci at which these gene copy number variants are located are shown in Fig. 1C. A waterfall diagram showing the tumor mutations of these ten genes was constructed (Fig. 1D). Somatic mutations in these genes were found in 32 of 289 patients, with the highest mutation rate being *LRPPRC*.

### Construction of a prediction index for disulfidptosis-associated lncRNAs

**Figure 2:**
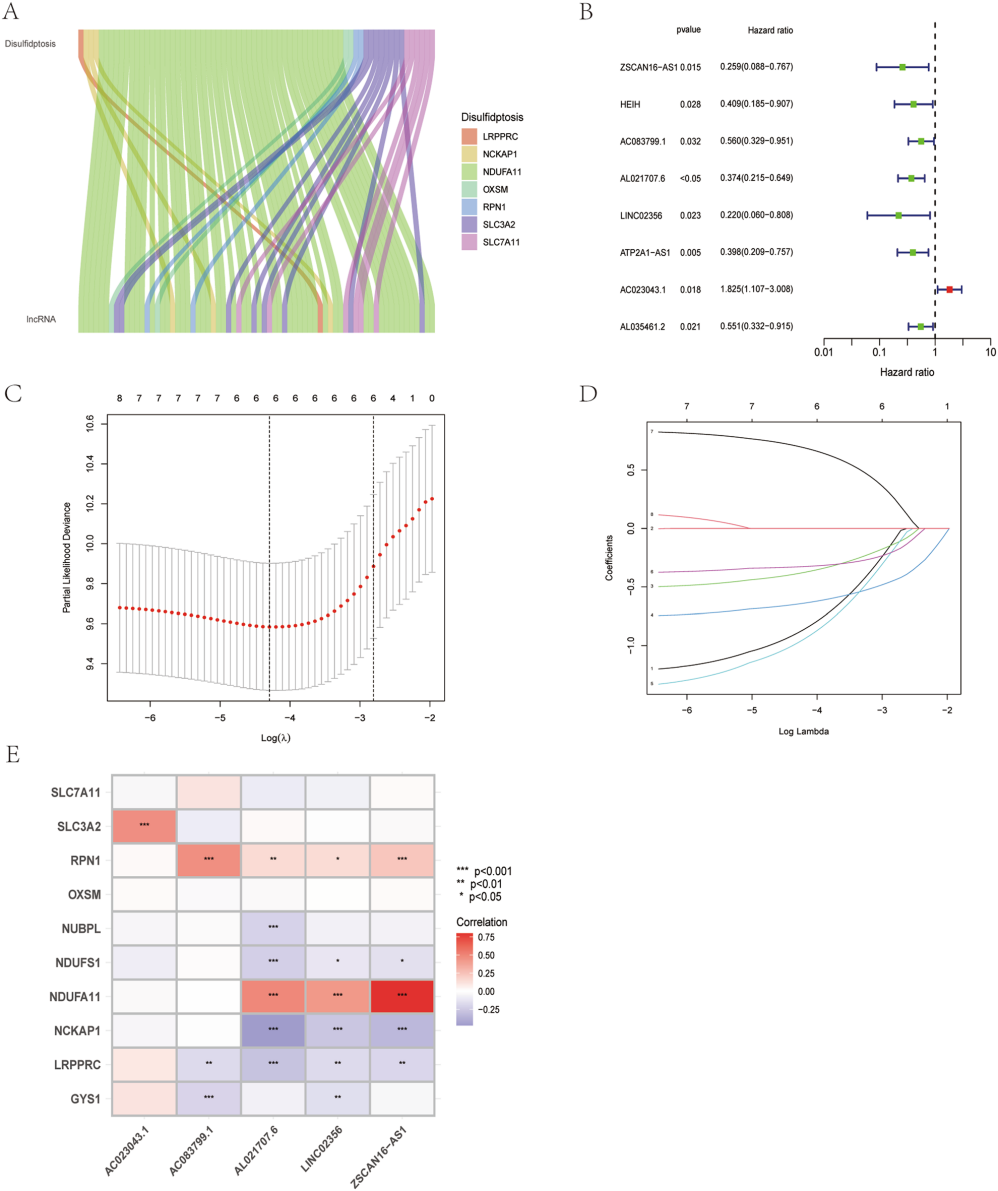
Identification of the signature of disulfidptosis-related lncRNA. (**A**) Sankey diagram of lncRNAs co-expressed with disulfidptosis-related genes. (**B**) Forest plots of 8-lncRNA screening by COX regression related with disulfidptosis. (**C**,**D**) Lasso regression of the DLI of 5-lncRNA. (**E**) Heatmap shows the co-expression relationship between disulfidptosis-related lncRNA index and disulfidptosis-related genes.

Spearman correlation analysis was performed to identify 71 lncRNAs associated with disulfidptosis-related genes (Fig. 2A). Subsequently, univariate COX regression analysis was conducted on the lncRNAs to assess their prognostic relevance. Based on the results, eight differentially expressed lncRNAs with prognostic significance were further screened and selected. Among the differentially expressed lncRNAs, one was determined as a risk factor and the remaining seven were identified as protective factors. We integrated transcriptomic data from TCGA-CESC datasets with clinical data, which generated training cohort and a testing cohort. No statistical difference in clinicopathological characteristics between the training and testing cohorts (Table 1). The LASSO-COX regression analysis was applied to the training set (eight candidate lncRNAs) to determine the optimal prediction score. Based on the optimal penalty parameter (λ) determined using the LASSO model, five lncRNAs were finally constructing the DLI. Cvfit curves and λ curves are shown in Fig. 2C,D. The expression differences of the five genes between the tumor tissues and normal tissues are shown in Supplementary Fig. S1. *AC023043.1*, *AC083799.1*, and *AL021707.6* were up-regulated in tumor tissues, whereas *LINC02356* and *ZSCAN16-AS1* were up-regulated in normal tissues. We showed the association of these five lncRNAs with disulfidptosis-related genes in Fig. 2E, and the results indicated different degrees of regulation and correlation between the five lncRNAs and disulfidptosis-related genes. Particularly, the five lncRNAs showed a positive relationship with *SLC3A2*, *RPN1*, and *NDUFA11* and a negative relationship with *NUBPL*, *NDUFS1*, *NCKAP1*, *LRPPRC*, and *GYS1*.

**Table 1:** Clinical characteristics of CC patients involved in the study.

		Total N = 304	Testing set n = 152	Training set n = 152	P-value
Age	≤ 65	269 (88.49%)	135 (88.82%)	134 (88.16%)	1
Age	> 65	35 (11.51%)	17 (11.18%)	18 (11.84%)	
Grade	G1	18 (5.92%)	7 (4.61%)	11 (7.24%)	0.5572
Grade	G2	135 (44.41%)	70 (46.05%)	65 (42.76%)	
Grade	G3	118 (38.82%)	59 (38.82%)	59 (38.82%)	
Grade	G4	1 (0.33%)	0 (0%)	1 (0.66%)	
Grade	Unknow	32 (10.53%)	16 (10.53%)	16 (10.53%)	
Stage	Stage I	162 (53.29%)	74 (48.68%)	88 (57.89%)	0.3226
Stage	Stage II	69 (22.7%)	33 (21.71%)	36 (23.68%)	
Stage	Stage III	45 (14.8%)	27 (17.76%)	18 (11.84%)	
Stage	Stage IV	21 (6.91%)	12 (7.89%)	9 (5.92%)	
Stage	Unknow	7 (2.3%)	6(3.95%)	1 (0.66%)	
T	T1	140 (46.05%)	68 (44.74%)	72 (47.37%)	0.3438
T	T2	71 (23.36%)	35 (23.03%)	36 (23.68%)	
T	T3	20 (6.58%)	7 (4.61%)	13 (8.55%)	
T	T4	10 (3.29%)	7 (4.61%)	3 (1.97%)	
T	Unknow	63 (20.72%)	35 (23.03%)	28 (18.42%)	
M	M0	116 (38.16%)	59 (38.82%)	57 (37.5%)	0.8219
M	M1	10 (3.29%)	6 (3.95%)	4 (2.63%)	
M	Unknow	178 (58.55%)	87 (57.24%)	91 (59.87%)	
N	N0	133 (43.75%)	67 (44.08%)	66 (43.42%)	0.5919
N	N1	60 (19.74%)	27 (17.76%)	33 (21.71%)	
N	Unknow	111 (36.51%)	58 (38.16%)	53 (34.87%)	

### Characteristics of DLI

**Figure 3:**
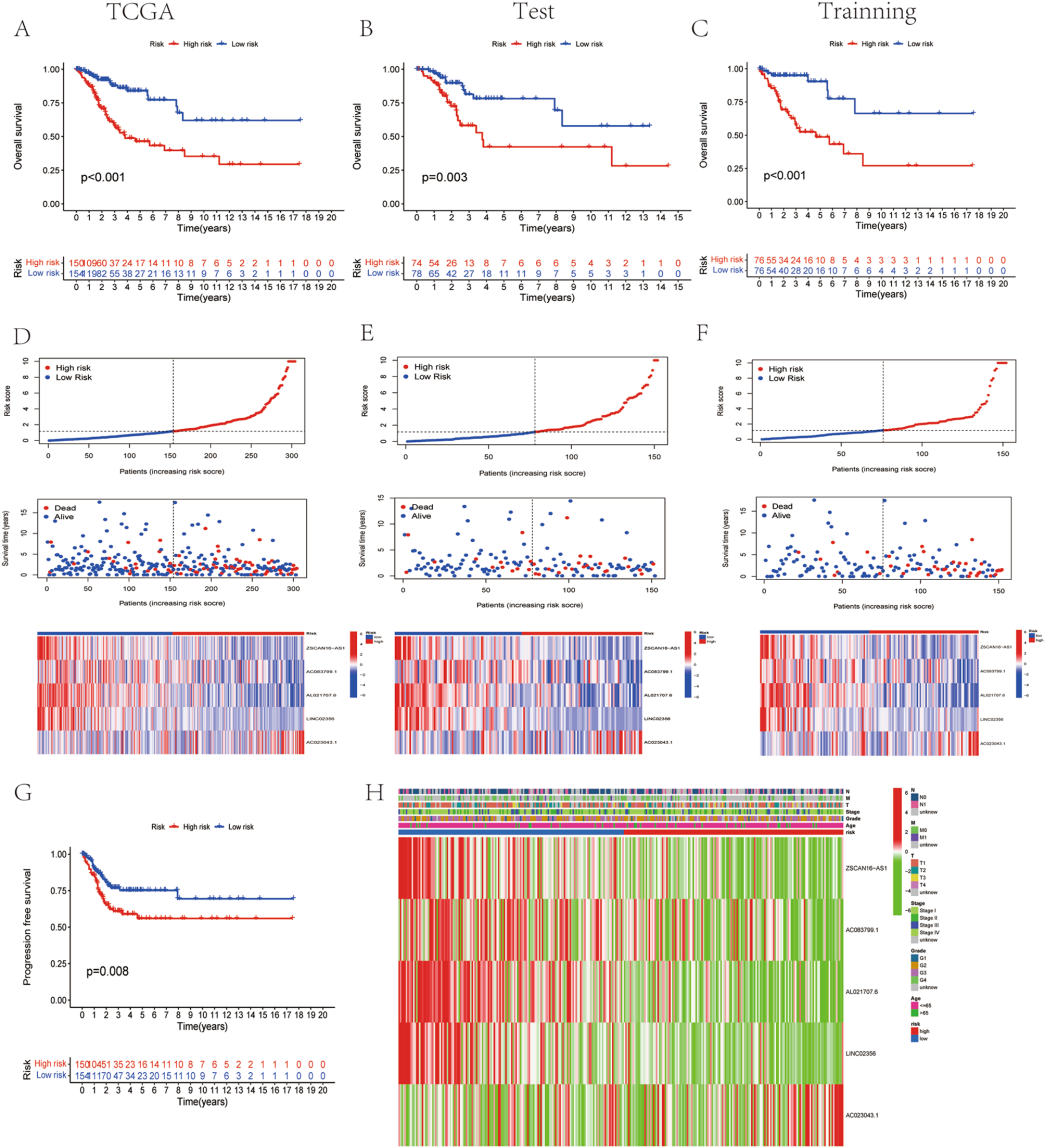
Characteristics of disulfidptosis-related lncRNA index. (**A**–**C**) The OS of TCGA cohort, the testing cohort, and the training cohort. (**D**–**F**) Risk characteristics between high- and low- risk (DLI) groups in TCGA cohort, the testing cohort, and the training cohort. (**G**) The PFS survival analysis in TCGA cohort. (**H**) Heatmap of expression in five lncRNAs in TCGA and distribution of clinical features.

To assess the predictive potential of the DLI, we performed prognostic analyses in three cohorts: TCGA cohort, training cohort, and testing cohort. Patients in these cohorts were categorized into two groups, namely, low- and high-DLI groups, according to the median score of the DLI. Kaplan–Meier analysis of OS analysis revealed that patients in the low-DLI group exhibited a better prognosis than these in high-DLI group across all three cohorts (Fig. 3A–C). In all three cohorts, patients in the high-DLI group had an increased risk of death when compared with those in the low-DLI group (Fig. 3D–F). Furthermore, PFS was also evaluated in patients with CC, which yielded consistent results with the predicted outcome of OS, showing improved PFS outcomes in the low-DLI group (Fig. 3G). Risk grouping and clinical characteristics of all CC cases are shown in Fig. 3H. As shown in the figure, the lncRNA AC023043.1 showed high expression in patients of the high-DLI group and was a risk factor, whereas the other four lncRNAs showed low expression and were protective factors.

### Assessment of DLI predictive capability

**Figure 4:**
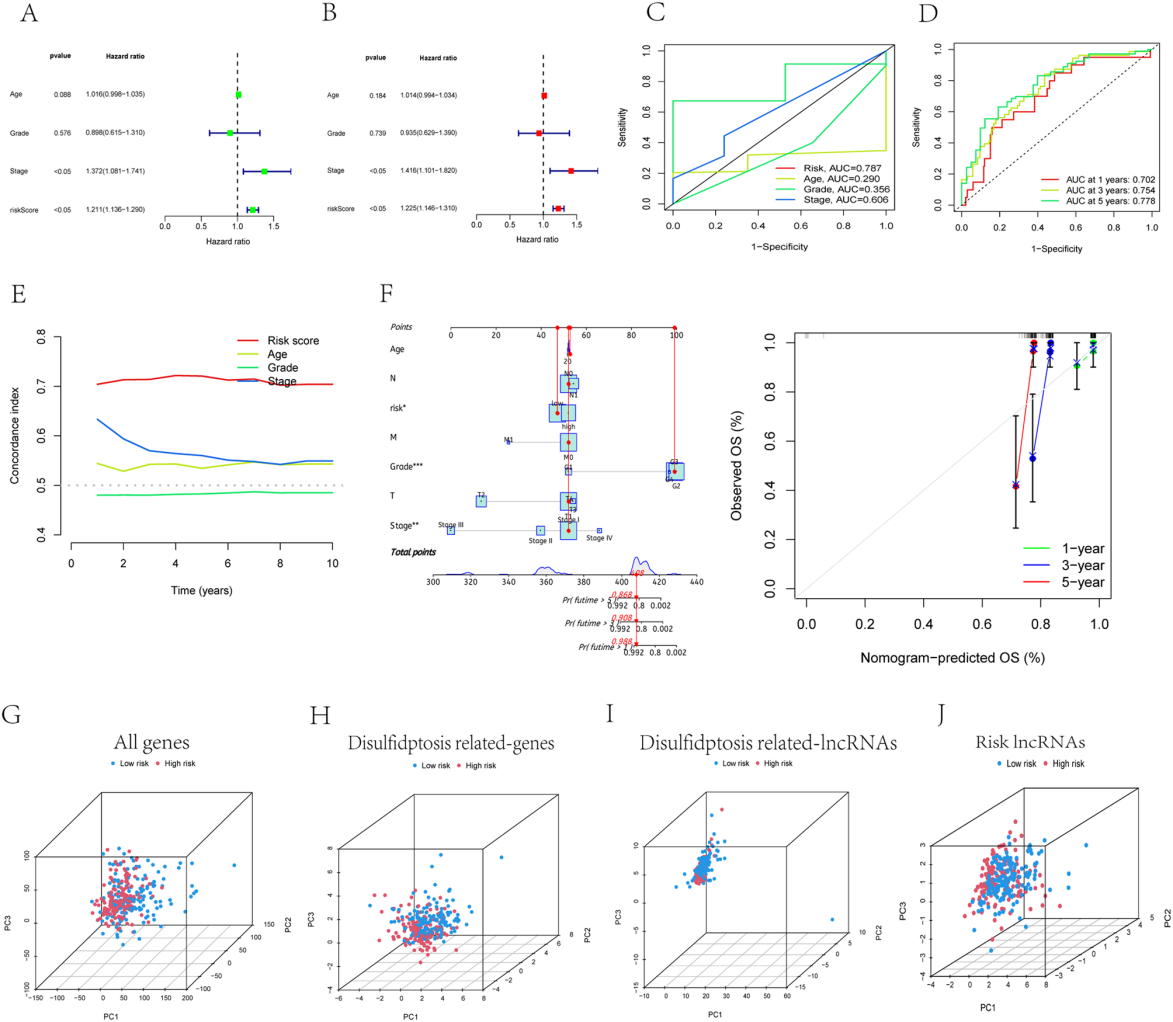
Validation of the disulfidptosis-related lncRNA signature. (**A**,**B**) The disulfidptosis-related lncRNA signature was shown to be an independent risk factor for patients' overall survival in TCGA. (**C**) The AUC showed that index (risk score) was an independent predictor compared with other clinicopathological signatures. (**D**) The C-Index of the DLI (risk score) was higher than other clinicopathological signatures. (**E**) The DLI could be used as an independent predictor to predict the OS of 1-, 3-, 5 years. (**F**) The nomogram of DLI combined with other clinical features. (**G**) PCA of all genes. (**H**) PCA of disulfidptosis-related genes. (**I**) PCA of disulfidptosis-related lncRNAs. (**J**) PCA of DLI.

To assess the independence and accuracy of the DLI, we performed univariate and multivariate Cox regression analyses incorporating patient age, sex, and tumor grade to determine independent prognostic factors (Fig. 4A,B). The hazard ratio of the risk score was statistically significant (P < 0.05). These r analyses indicated that the prognostic predictive power of the DLI was independent of other clinical characteristics. Furthermore, the ROC curve (Fig. 4C) demonstrated that DLI was a more accurate predictors than other clinical characteristics (area under the curve [AUC] = 0.787). The predictive power of survival 1 years (AUC = 0.702), 3 years (AUC = 0.754), and 5 years (AUC = 0.778) was excellent (Fig. 4D), The C-index curves further supported these findings, with C-index values being greater than 0.7 (Fig. 4E). We believed that combined DLI and other clinical characteristics (age, grading, staging, T, N, M) is a more convenient predictive tool and has stronger predictive power in clinical applications. So we calculated nomoscore and plotted nomogram (Fig. 4F). The nomoscore showed a significant increase in the predictive confidence for 1-, 3- and 5-year survival rates after combining clinical features (0.988, 0.908, and 0.898, respectively). To validate the spatial grouping performance of risk scores for patients with CC and visualize the discriminating ability and power of DLI among patients, we conducted PCA for all four gene groups (Fig. 4G–J). The results indicated that DLI could more accurately determine the risk cutoff for patients than the other gene sets.

### Gene ontology analysis and gene set enrichment analysis

**Figure 5:**
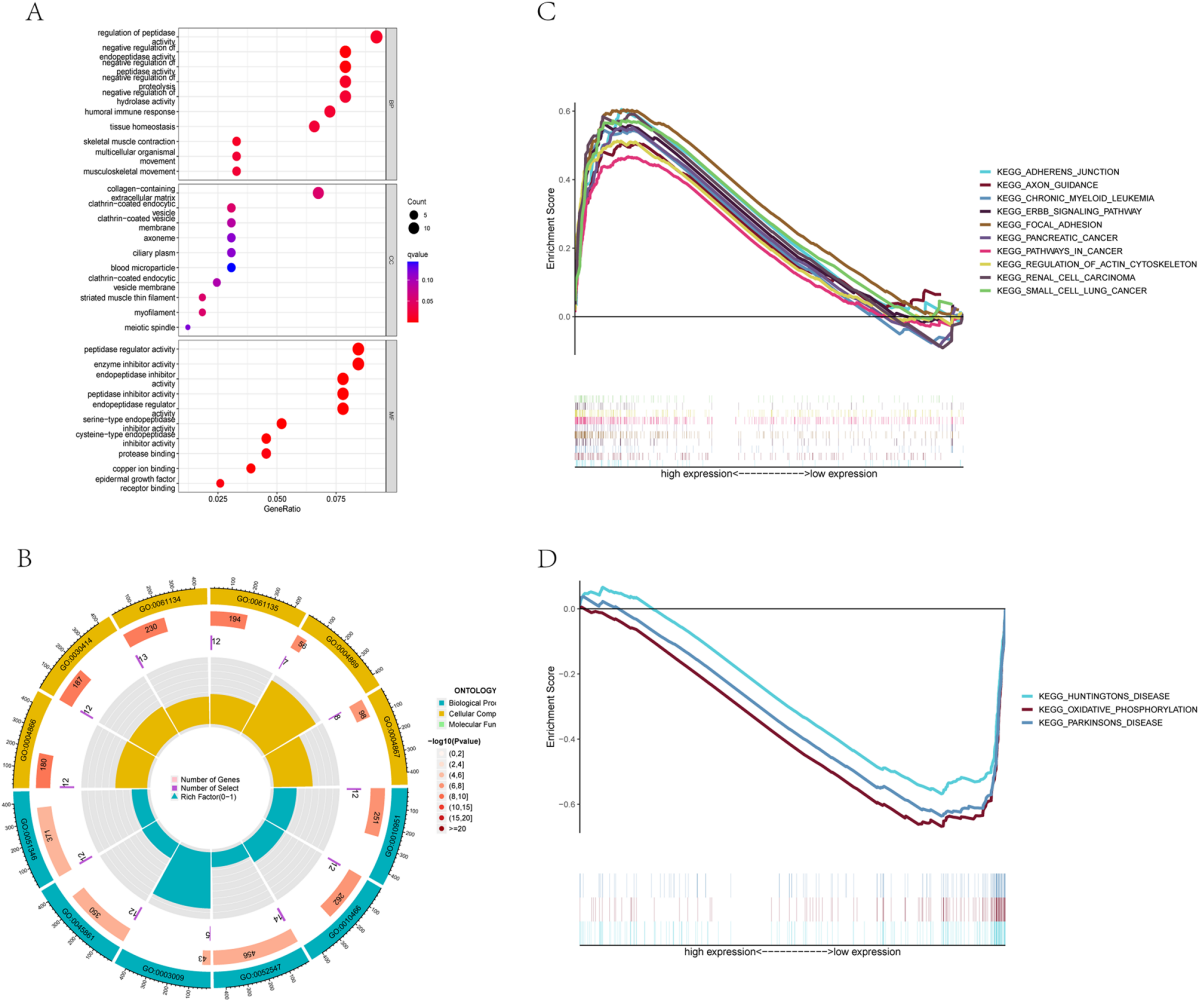
GO and GSEA analysis. (**A**) The bubble diagram of GO analysis. (**B**) The circle graph of GO analysis. (**C**) GSEA analysis of the high DLI group. (**D**) GSEA analysis of the low DLI group.

Additionally, we performed gene ontology (GO) analysis on the differentially expressed genes between the low-DLI and high-DLI groups. The analysis revealed that the differentially expressed genes were enriched mainly in the regulation of peptidase activity and function (Fig. 5A,B). Furthermore, gene set enrichment analysis (GSEA) demonstrated that the high-DLI group was enriched mainly in malignant and pan-cancer-related pathways as well as cell adhesion (Fig. 5C). By contrast, the low-DLI group was functionally enriched mainly in neurological disorders and biological oxidation (Fig. 5D).

### Immune infiltration for risk scores and prediction of immunotherapy

**Figure 6:**
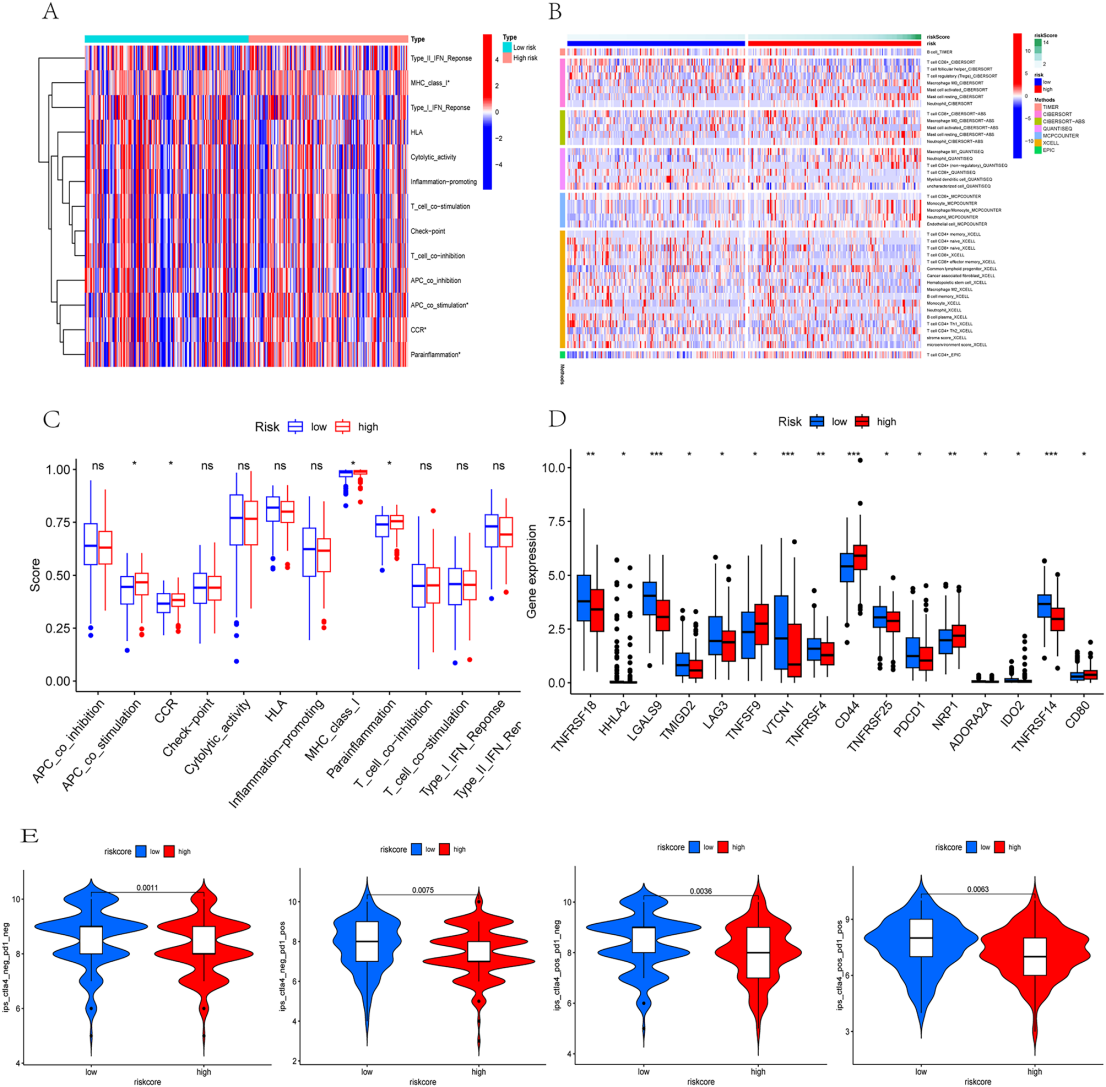
Immune infiltration analysis of the signature. (**A**) Heatmap showed immune cells and immune function between high and low risk groups in all samples. (**B**) Heatmap of immune penetration based on TIMER, CIBERSORT, CIBERSORT-ABS, QUANTISEQ, MCPCOUNTER, and XCELL algorithms. (**C**) Differences in immune function between high- and low-DLI groups. (**D**) Differences in immune checkpoints between high- and low-DLI groups. (**E**) Differences in IPS between high- and low-DLI groups.

The immune function of the two risk groups is presented in Fig. 6A. Quantification of the differences is shown in Fig. 6C, indicating that among the four statistically different immune functions (antigen-presenting cell [APC] co-stimulation, chemokine receptor [CCR], major histocompatibility complex [MHC] class I presentation, and para-inflammation), the high-DLI group showed enrichment in immune functions when compared with the low-DLI group. Additionally, we assessed immune cell infiltration between the low-DLI and high-DLI groups (Fig. 6B), and observed higher infiltration of CD4^+^T cells and CD8^+^T cells in the high-DLI group. Furthermore, we analyzed the differences in immune checkpoints between the two groups (Fig. 6D) and observed that the TNF family was highly expressed in the low-risk group, which may be one of the reasons for tumor progression inhibition. By contrast, *VTCN1* was highly expressed in the low-risk group when compared with that in the high-risk group, which requires further investigation. To obtain a prediction of the effectiveness of immunotherapy for CC, we calculated the immune prognostic score (IPS) for patients in the high-risk and low-risk groups. We discovered that regardless of the positive or negative status of CTLA4 and programmed death ligand 1 (PD-L1), the IPSs were lower in the high-risk group than in the low-risk group, indicating a potentially better response to immunotherapy (Fig. 6E).

### Tumor mutational burden differences in DLI

**Figure 7:**
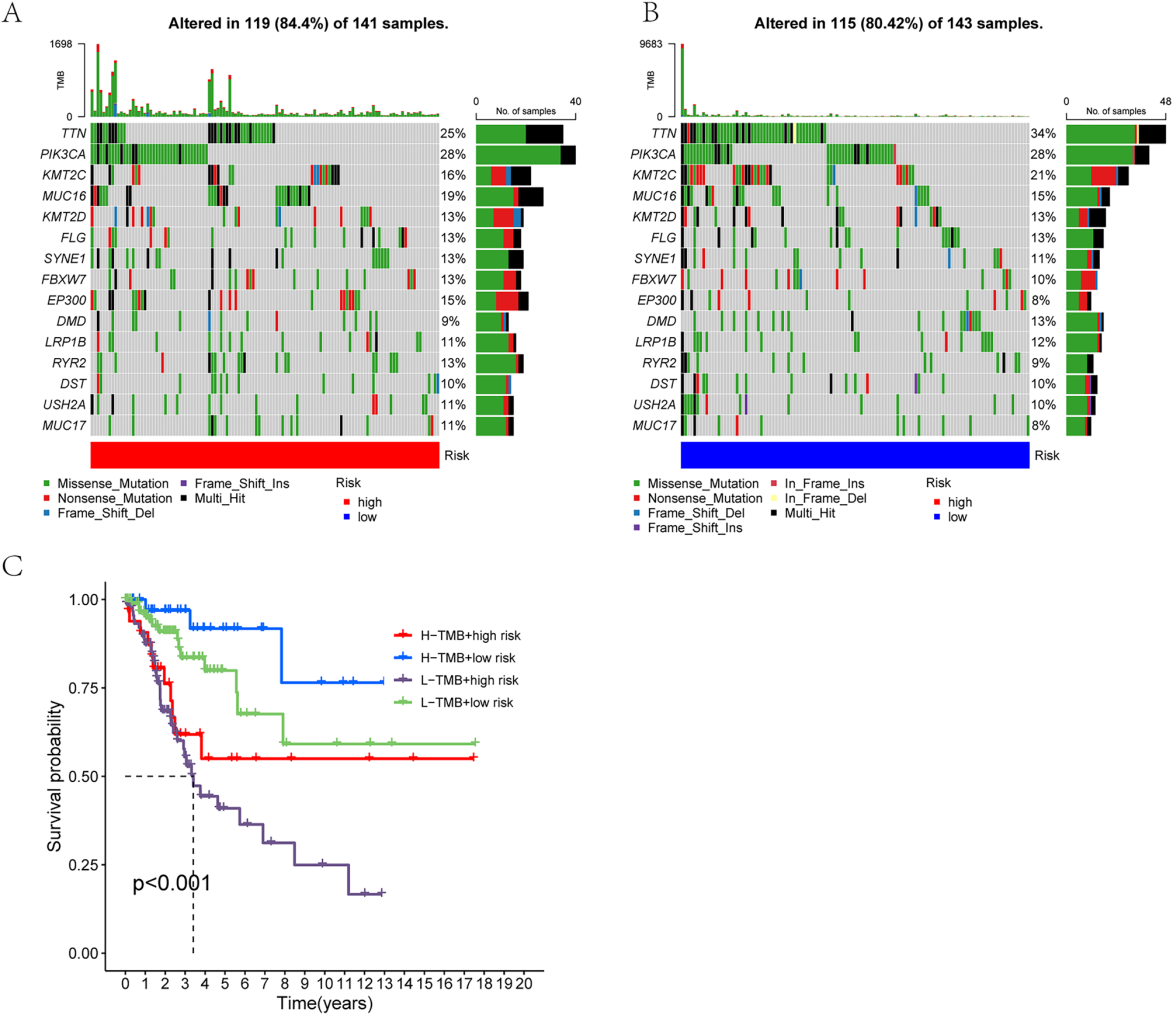
Relationship between DLI and tumor mutation burden. (**A**,**B**) Waterfall plots of somatic mutation characteristics in the high-DLI and low-DLI groups. (**C**) K–M survival curves between the four groups.

Tumor mutational burden (TMB) is crucial for tumor prognosis. The mutations in different DLI groups are depicted in Fig. 7A,B. While the mutation rate was slightly higher in the high-DLI group than in the low-DLI group, the difference was no statistically significant. Notably, the number of mutations in *TTN* (Titin gene) and *KMT2C* was significantly higher in the low-DLI group than in the high-DLI group. While further analyzing the predictive role of TMB on OS, we observed that the high-mutation group combined with the low-DLI group had the most favorable prognosis, whereas the low-mutation group combined with the high-DLI group had the worst prognosis (Fig. 7C).

### Prediction of chemotherapy drug sensitivity by DLI

**Figure 8:**
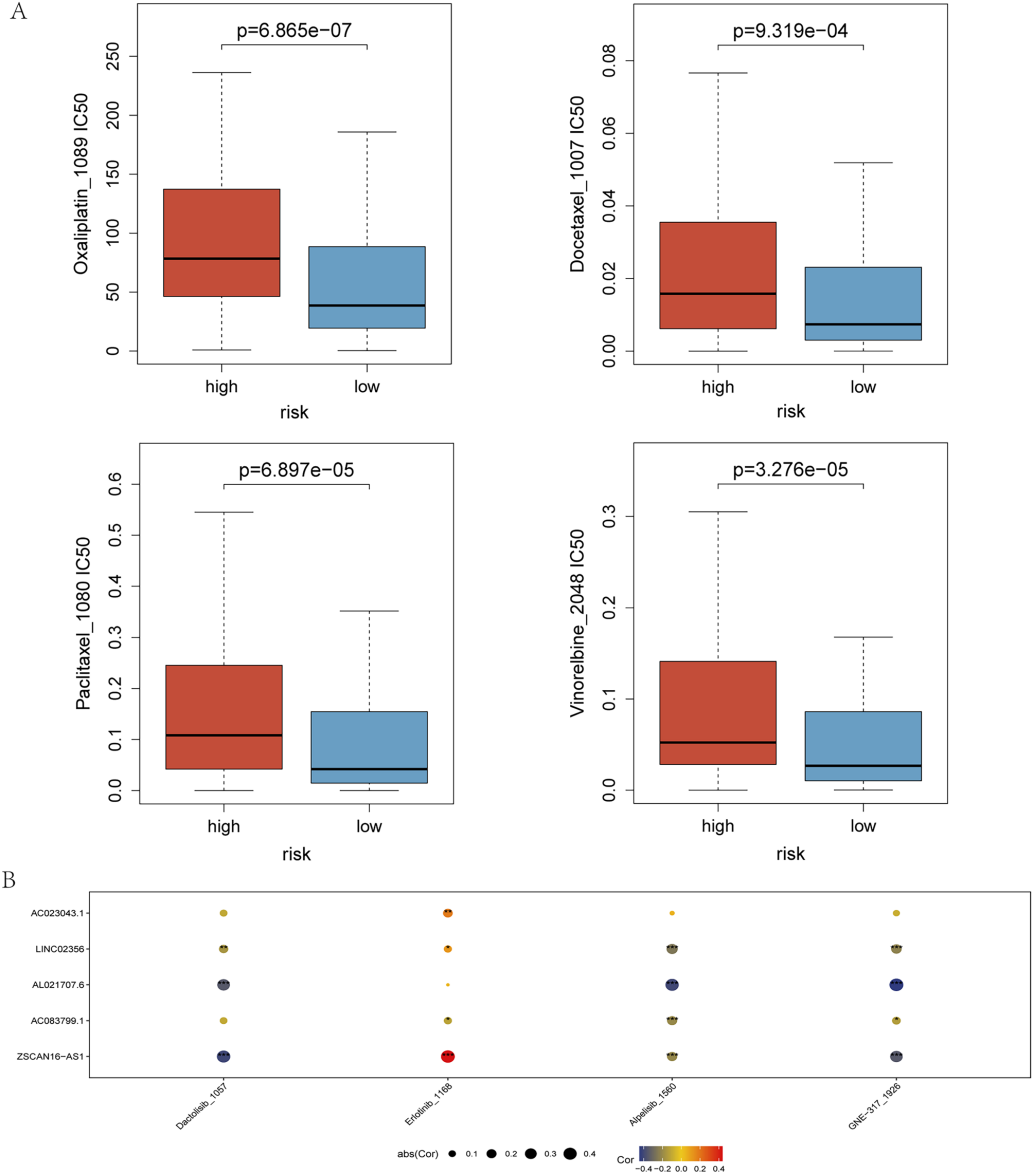
Chemotherapy drug sensitivity prediction for DLI. (**A**) Differences in sensitivity of commonly used chemotherapeutic drugs for CC. (**B**) Chemotherapeutic drug prediction with correlation to five lncRNAs in DLI.

Chemotherapy is an important treatment for CC. To predict the differences in chemotherapeutic drugs sensitivity among patients in different DLI groups, we calculated the IC_50_ values of various chemotherapeutic drugs in different risk groups using the Genomics of Drug Sensitivity in Cancer (GDSC) database. The results demonstrated that the IC_50_ values for all commonly used chemotherapeutic agents in CC were higher in the high-DLI group than in the low-DLI group. Specifically, the sensitivity to chemotherapeutic drugs was higher in the low-DLI group than in the high-DLI group (Fig. 8A). Furthermore, we identified four chemotherapy drugs (oxaliplatin, docetaxel, paclitaxel, and vinorelbine) for use in CC. The chemotherapeutic drugs that correlated with the five lncRNAs of the construct score were screened for future research on CC treatment. Figure 8B shows a bubble plot illustrating the correlation between gene expression and chemotherapeutic drugs, where the size and color of the bubbles represent the magnitude of positive and negative correlations, respectively. *AL021706.6* expression showed a negative correlation with the IC_50_ values of Dactolisib, Alpelisib and GNE-317, while *ZSCAN16-AS1* showed a positively associated with Erlotinib, and a negatively associated with Dactolisib and GNE-317.

## Discussion

ncRNAs, especially lncRNAs, affect CC progression through the cell death pathway and sensitivity to radiotherapy^21,22^. However, given the limited treatment options and poor prognosis of locally advanced and intermediate-stage CC^23^. The discovery of novel predictive targets and exploration of potential therapeutic agents are of utmost importance. Disulfidptosis, a newly discovered mode of cell death, has not yet been extensively studied in CC. Nonetheless, we believe that disulfidptosis involves related genes regulated by ncRNAs. Given our focus on lncRNAs, we screened publicly available databases to explore disulfidptosis-related lncRNAs that could serve as predictive targets.

In the present research, we investigated the expression of disulfidptosis-related genes in CC and observed that these ten genes had upregulated expression in tumor tissues. Consequently, we identified lncRNAs associated with upstream-elements and used a machine learning approach to screen for five lncRNAs differentially expressed lncRNAs with prognostic and predictive ability. These five lncRNAs were used to construct a DLI for CC. This index demonstrated the ability to predict the risk of death, OS, and PFS in patients with CC, with better outcomes observed in the low-DLI group. We conducted a literature survey on the five lncRNAs that comprising the index and discovered that three of the lncRNAs had been previously reported in studies on other cancer types. For instance, silencing of *ZSCAN16-AS1* inhibits hepatocellular carcinoma (HCC) cell proliferation, migration and invasion, as well as acceleration of HCC cell apoptosis^24^. However, in our study, *ZSCAN16-AS1* was highly expressed in the low-DLI group, a finding that is inconsistent with its expression in HCC. We believe this inconsistency is attributed to the heterogeneity of tumors. However, this finding should be confirmed in future studies on CC apart from elucidating the underlying mechanism. *AC083799.1* as well as *AC023043.1* are part of predictive models for the prognosis of endometrial cancer^25^ and kidney cancer^26^, respectively. Nevertheless, *AL021707.6* and *LINC02356* have not been reported yet, presenting a direction for future research in CC.

We conducted further validation to assess the independence, accuracy, and specificity of the DLI in prognostic prediction for CC. Independent prognostic analysis verified that the index could serve as a prognostic factor independent of other clinical characteristics. The ROC curve demonstrated that the diagnostic accuracy and predictive accuracy of the index were superior to those of other clinical factors, while the C-index curve indicated a higher specificity than that for other clinical factors. Furthermore, the risk score derived from the index was more widely applicable and provided a clearer definition of patient management stratification than other genetic characteristics. The Nomoscores, calculated in conjunction with other clinical factors, improved the accuracy of the prognostic assessment for patients. These validations collectively indicate that the DLI exhibits superior independence, accuracy and specificity and can be used as a target for prognostic prediction in CC.

The immune environment, which is the most critical part of the tumor microenvironment, plays a crucial role in the regulating disease progression and response to anticancer therapy^27,28^. Currently, programmed death ligand 1 (PD-L1) expression is utilized in clinical practice to identify patients with CC who may benefit from immune checkpoint inhibitor therapy. However, recent information suggests that PD-L1 may not be a completely reliable biomarker for patient selection^29^. Therefore, we conducted further analysis of the immune microenvironment between the high- and low-DLI groups based on the index. Our findings revealed that immune functions (APC-co-stimulation, CCR, MHC-class-I and parainflammation) were enriched mainly in the high-DLI group. Conversely, several algorithms for immune infiltration indicated a greater enrichment of CD4^+^ T cells and CD8^+^ T cells in the low-DLI group than in the high-DLI group. This disparity can be explained by the prolonged presence of tumor antigens and inflammatory stimuli in the high-DLI group, leading to T-cell exhaustion^30^, and weakened tumor-killing ability, thus resulting in a worse prognosis. Based on these findings, we believe that strategies aimed at alleviating T-cell exhaustion and enhancing effector T-cell function hold promise as potential therapeutic directions for improving the prognosis of patients with CC.

In terms of immune checkpoint expression, *TNFRSF18*, *TNFRSF4*, *TNFRSF24*, *TNFRSF14* were significantly upregulated in the low-DLI group. The tumor necrosis factor (TNF), TNF receptor (TNFR), and TNF/TNFR superfamily (TNFSF/TNFRSF) include 29 receptors and 19 ligands that play a crucial role in controlling cellular processes. Communication routes mediated by TNFSF and TNFRSF are essential for various developmental, homeostatic, and stimulus-responsive processes in vivo^31,32^. Members of the TNF family have been shown potential as novel targets for tumor immunotherapy^33^. *CD44,* another important clinical target, is commonly regarded as a cancer stem cell (CSC) marker in various malignancies. High expression *of CD44* significantly upregulate stumorigenic processes such as cell proliferation, metastasis, invasion, migration, and stemness^34^. Our study revealed upregulated *CD44* expression in the high-DLI groups, consistent with the findings of previous studies. *VTCN1* is also a well-reported immunotherapeutic target that promotes invasive metastasis in multiple cancer types including ovarian cancer^26^. In CC, *VTCN1* expression facilitates the immunosuppressive microenvironment by promoting the production of IL-10 and transforming growth factor(TGF)-β1, contributing to the progression of cervical carcinogenesis^35^. However, in our study, we observed the elevated *VTCN1* expression in the low-DLI group, which may attributed to the complex microenvironment of the tumor, and this finding should be validated in further investigations. Regarding the prediction of immunotherapy efficacy, IPS scores were calculated for the two DLI groups. The results indicated that the IPS score of the low- DLI group was higher than that of the high-DLI group in all four gene groups, suggesting that the low-DLI group has higher immunogenicity, while the high-DLI group may receive more benefit from immunotherapy strategies involving CTLA-4 blockers and PD-L1 blockers. These findings suggest that the index could serve as a reference for patient selection of immunotherapy regimens.

TMB is associated with clinical response to immune checkpoint blockade therapy in certain tumors^36,37^. Our study suggested no significant difference in total TMB between the high-DLI and low-DLI groups. However, *TTN* exhibited a considerably higher mutation frequency in the low-DLI group than in the high-DLI group, making it the gene with the largest difference in mutation frequency. *TTN* mutations are identified as prognostic factor in a variety of diseases, although their effect differs among different cancer types. For example, *TTN* mutations are positively associated with prognosis in lung squamous cell carcinoma (LUSC)^38^, but the association may differ in thyroid cancer^39^. Survival analysis also demonstrated that high TMB combined with low-DLI resulted in the most favorable prognosis. Future studies should further investigate whether the improved prognosis is attributed to increased TTN mutations.

The sensitivity of chemotherapy in patients with CC notably affects prognosis. Simultaneous chemotherapy and radiotherapy (concurrent chemoradiation therapy) plus brachytherapy is the standard-of-care treatment for CC beyond locally advanced stages^40,41^.The recommended chemotherapy regimens involve platinum-based agents, paclitaxel or docetaxel used in combination with platinum-based agents^42^. However, platinum drugs, which are non-specific chemotherapeutic agents, often cause severe systemic adverse reactions, and the recurrence and distant metastasis rates of CC after chemotherapy remain high at 40%^43^. Therefore, other chemotherapeutic agents have been extensively studied in CC treatment. Clinical trials investigating the use of vincristine for CC treatment have also been conducted^44^. Our findings indicated elevated IC_50_ values for oxaliplatin, docetaxel, paclitaxel, and vincristine in the high-DLI group, suggesting that the low-DLI group exhibited better chemotherapy sensitivity than the high-DLI group. This can be directly applied to the clinical selection of commonly used chemotherapeutic agents.

Furthermore, our study derived novel findings by refining the prediction of the expression of the five lncRNAs in DLI in relation to the sensitivity to targeted drugs. For instance, Dactolisib, a dual PI3K and mTORC1/2 inhibitor has shown potential benefits in CC treatment^45^. Erlotinib treatment sensitizes CSCs to paclitaxel treatment in vitro and in vivo^46^, Alpelisib demonstrates antitumor effects and enhances cisplatin efficacy through the PI3K/AKT pathway in *PIK3CA* mutant CC cells^47^. The expression of *AL021707.6* as well as *ZSCAN16-AS1* showed interesting correlation with the IC_50_ of these drugs, indicating a new direction in the study of targeting drugs in CC.

Combining the aforementioned predictions, we conclude that the risk classification of patients based on the risk scoring model constructed by disulfidptosis-related lncRNA provides valuable insights. We developed a DLI consisting of five lncRNAs. Of these five lncRNAs, *ZSCAN16-AS1*, *AC083799.1*, *AL021707.6*, and *LINC02356* can be used as protective factors, while *AC023043.1* can be used as a risk factor. The high-DLI group may exhibit increased sensitivity to immunotherapy, while the low-DLI group is more likely to benefit from chemotherapy. Clinically, detecting of the expression levels of these five lncRNAs can be used to predict the prognosis of patients with CC, guiding immunotherapy predictions, and aid the selection of chemotherapeutic agents and targeted drugs.

Our study still has some shortcomings. First, our study was based on a public database with limited sample size and lack of external data set validation. Secondly, we need to implement further experimental programs to confirm our study. In addition, the mechanism by which our model affects the prognosis of still needs further investigation.

## Conclusions

Our study defined five lncRNAs associated with disulfidptosis, and their index was shown to predict the prognosis of CC patients with independence, accuracy, and specificity, which could predict patient survival and facilitate clinical management of patients in a stratified manner combined with clinical characteristics. In addition, it could be used as a predictive target for the prognosis and treatment of CC, as well as a guide for the sensitivity of patients to immunotherapy and chemotherapy.

## Supplementary Information

Supplementary Figure S1.

## Data Availability

The RNA sequencing profiles are able to be gained from The Cancer Genome Atlas (TCGA) (https://portal.gdc.cancer.gov/). Further inquiries can be directed to the corresponding author.

## Abbreviations

CESC: Cervical squamous cell carcinoma and endocervical adenocarcinoma
CC: Cervical cancer
LncRNA: Long non-coding RNA
CNV: Copy number variation
SNV: Single nucleotide variation
TTN: Titin gene
HCC: Hepatocellular carcinoma
CSC: Cancer stem cell
TCGA: The Cancer Genome Atlas
LASSO: Least absolute shrinkage and selection operator
ROC: Receiver operating characteristic curve
OS: Overall survival
PFS: Progression-free survival
GSEA: Gene set enrichment analysis
HR: Hazard ratio
K-M: Kaplan–Meier
GO: Gene ontology
TMB: Tumor mutation burden
IPS: Immunophenoscore
