# Supplementary material for: Disulfidptosis-associated LncRNAs index predicts prognosis and chemotherapy drugs sensitivity in cervical cancer
Source: Sci Rep. 2023 Aug 1;13:12470. doi: 10.1038/s41598-023-39669-3 (PMC10394072; doi:10.1038/s41598-023-39669-3)
Supplement: Supplementary file 1 — Supplementary Figure S1. [file 41598_2023_39669_MOESM1_ESM.docx]

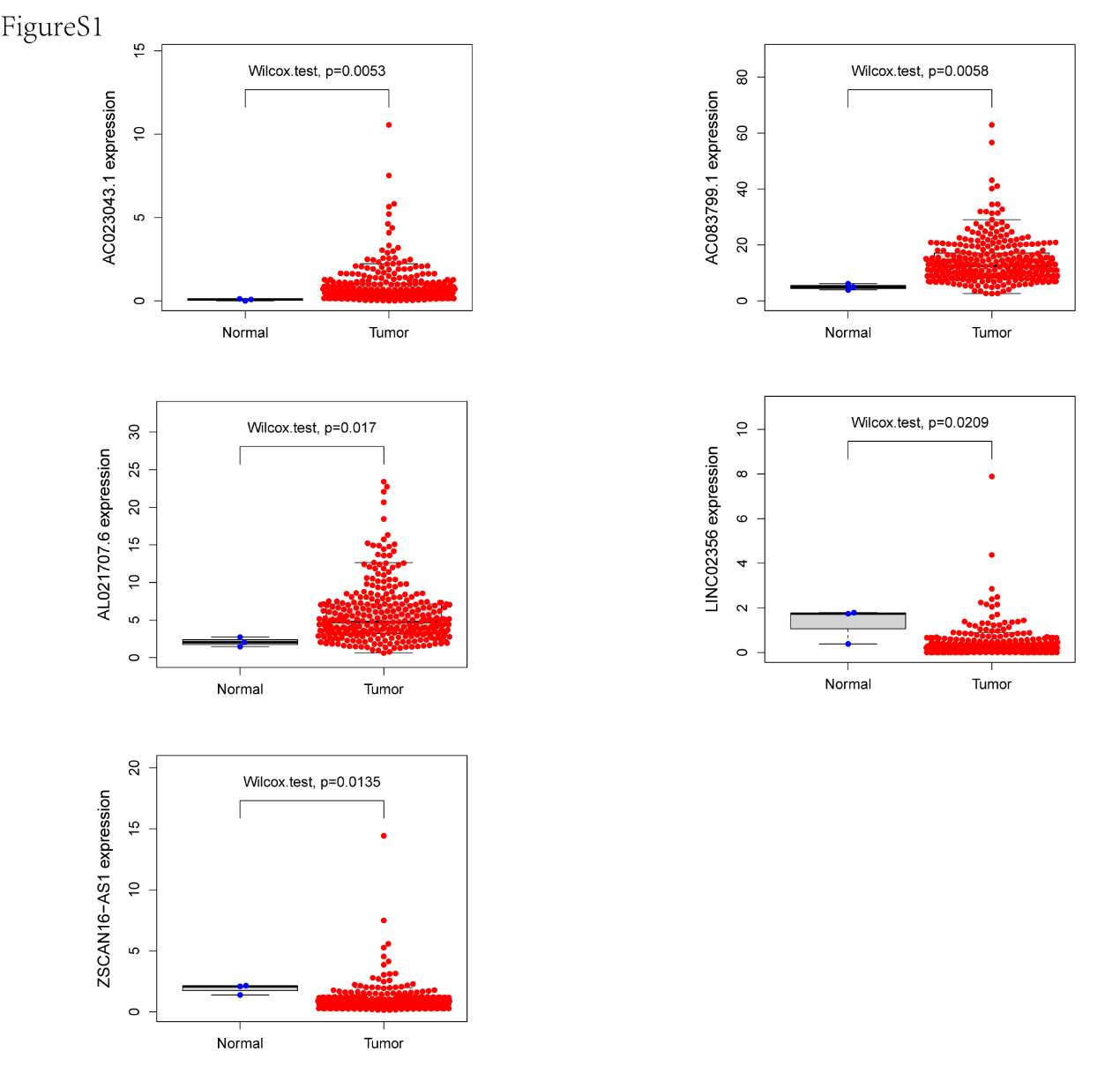


FigureS1: Differential expression of five lncRNAs between cervical cancer tissues and normal tissues.
